# Editorial: Phenomics

**DOI:** 10.3389/fpls.2018.00678

**Published:** 2018-05-23

**Authors:** Marcos Egea-Cortines, John H. Doonan

**Affiliations:** ^1^Genética Molecular, Instituto de Biotecnología Vegetal, Universidad Politécnica de Cartagena, Cartagena, Spain; ^2^National Plant Phenomics Centre, Aberystwyth University, Aberystwyth, United Kingdom

**Keywords:** phenomics, artificial vision, RGB data, RGB image analysis, multispectral imaging

Developments in high throughput molecular technologies, from DNA sequencing to metabolite analysis and proteomics, have opened up new and previously undreamt of vistas in biology. Previously, one often had to make a difficult choice between longitudinal and cross-sectional studies but, with these highly scalable technologies, the number of individuals that can be screened has increased so dramatically that temporal studies are possible on whole populations. The technologies tend not to be specific to a given species, allowing us to sample the entire tree of life. One consequence of this technological explosion is that measurement of phenotypic traits for large populations over developmental time and in response to environmental variable has become highly desirable, if not a necessity (Houle et al., 2010). In this context, the development of new technologies to obtain reliable phenotypic data is a pre-requisite to approaching the overall challenge. As compared to the genotype (or even the proteome), the phenotype is highly dimensional to the extent that measuring all possible phenotypic traits is not feasible. However, the concept of “phenomics” has been proposed to cover sets of technologies devised to obtain phenotypic data in an analogous way to ‘omics associated with the various molecular technologies. Phenomics therefore includes a vast array of approaches that, in most cases, include some sort of automatic sampling or non-invasive methods to obtain repeated sampling from an individual or population.

The general requirement for reproducibility is an additional driver for phenomics. While commercial phenotyping platforms can be very powerful (Virlet et al., 2017), the economic aspects of purchasing and maintenance and the lack of flexibility (in what are emerging technologies) has fostered in-house developments (Navarro et al., 2012; Lou et al., 2014). In plant biology, growth conditions (the environment) play a key role in the final phenotype of a plant and having well-defined growth parameters is not yet the rule (despite what the material and methods section of a typical peer-reviewed research paper might imply). In this special topic on Phenomics, Negi et al. addressed reproducibility of growth conditions, developing a modified hydroponic system to test for phosphate deficiency on rice root traits. The digital nature of the data is a major advantage as it allows sharing and re-use, both key to the success of the other ‘omics technologies. An open-source software tool (Seedusoon) allows management of germplasm gathering together phenotypic and genetic data for a given accession (Charavay et al.).

Many of the non-destructive phenomic approaches rely on image analysis systems to acquire and process images. While the approach may seem straightforward, quantitative extraction of interesting features, such as intensity of image pixels, geometry of pixels or textures, remains challenging, and trade-offs between the ideal and the affordable are commonplace. For example, a key decision involves the type of camera used for data capture as that can limit the band width used to measure a given trait. This will have knock-on consequences, affecting the procedures used for image analysis (Navarro et al., 2016; Perez-Sanz et al., 2017). In the current edition, several publications t address issues associated with the analysis of a variety of plants using different image acquisition devices. Standard cameras including those found in smartphones perform image acquisition with an RED-GREEN-BLUE or RGB sensor. One study utilizes RGB images to determine wheat density at early stages of development (Liu et al.). There is an increasing number of publicly available libraries that facilitate image analysis (see Perez-Sanz et al., 2017 for a review). OpenCV, a widely used image processing library, underpinned development of SeedCounter (Komyshev et al.). This free Android App for mobile phone and pads, provides seed and grain morphometry under lab and field conditions, with much of the functionality of much more expensive equipment.

Stereo-vision is a long-established technique that uses two carefully positioned RGB cameras to capture 3-D information. Growth has been monitored in four species of tree seedling using the green channel and a stereo-vision approach (Montagnoli et al.). A regression model between the level of “greenness” and the real biomass obtained by destructive measures gave R values ranging between 0.67 for *Fagus sylvatica* and 0.95 for *Quercus ilex*, again showing actual differences between plants for a given setup. The interaction between canopy structure and photosynthesis has been studied by coupling 3-D reconstruction with gas exchange analysis showing that even complex traits such as 3-D structures can be related to photosynthesis efficiency (Burgess et al.).

The non-visible wavelengths can provide additional information on physiology and function. Thermal infrared imaging devices mounted on unmanned aerial vehicles (UAV) enables high throughput analysis of *Populus nigra* populations for dynamic responses to drought stress (Ludovisi et al.). Combined hyperspectral and thermal imaging of lettuce reveals how these plants adapt to multiple stresses (Simko et al.). Hyperspectral imaging has high information content and can measure several parameters simultaneously when calibrated. Thus, parallel analysis of chlorophyll a, chlorophyll b, total chlorophyll, and carotenoid in rice showed high correlation with hand measurements is 0.827–0.928 at the tillering stage, illustrating great potential to screen large populations (Feng et al.).

Using a combination of five non-invasive camera-based imaging units equipped with fluorescent, RGB Visible Near Infrared (VNIR), Short Wave Infrared and three dimensional imaging, Lyu et al. determined a total of 200 quantitative traits during leaf senescence. This illustrates the enormous potential of phenomic approaches to have a comprehensive understanding of biological variation.

High-throughput screening of combinations of traits is the immediate promise of phenomics and is further exemplified by the use of near-infrared reflectance spectroscopic (NIRS) to undertake a coordinated analysis of oil, protein, carbon, and nitrogen content in Arabidopsis seeds. As a result, a set of QTLs controlling these traits, and the variance component of genotype, culture, Genetic by Environment interaction, and residual effect have been determined (Jasinski et al.).

Image-based approaches can be compromised by the quality of the signal obtained. This is an ongoing problem common to many ‘omics technologies where assessment of quality plays a key role in downstream data analysis. Directly addressing this problem (Lobos and Poblete-Echeverría) developed software to assess the quality of spectral reflectance data. As spectral reflectance data are widely used to obtain crop performance indices such as NDVI, this type of exploratory data analysis is essential for evaluating data quality.

## Author contributions

ME-C wrote draft. JD corrected it and both agreed on final version.

### Conflict of interest statement

The authors declare that the research was conducted in the absence of any commercial or financial relationships that could be construed as a potential conflict of interest.
